# A perspective of ultra-processed food definitions and classification challenges

**DOI:** 10.1093/af/vfaf048

**Published:** 2026-01-23

**Authors:** Cody L Gifford

**Affiliations:** Department of Animal Science, University of Wyoming, Laramie, WY

**Keywords:** food processing classification, NOVA, minimally processed meat, further processed meat

Implications

- Several food processing definitions and classification systems exist as frameworks to highlight general similarities by processing level.
- The NOVA (not an acronym) classification system is the most popular for categorizing ultra-processed foods.
- Definitions and NOVA classification of ultra-processed foods (UPF) may result in variable interpretation to accurately classify foods, often leading to misclassification between studies.
- Careful consideration of concentration levels for nutrients and ingredients, purpose of ingredients in food products, understanding what ‘formulation’ refers to and micronutrient density when evaluating whether food products are UPF is necessary.
- Research opportunities exist to classify meat products based on nutrient density, ingredient evaluation and relative nutrients to reduce using an enhanced classification system that has increased specificity beyond the NOVA classification framework. Further evaluation of minimally processed and further processed meat products on chronic health risk is warranted.

## Introduction

Variability and inconsistency of adopted terminology to describe the level and degree of food processing persists. Published definitions of processing applied throughout the food industry are generally based on the degree of physical, nutritional or biochemical alteration from the original whole food, often including the inclusion of additional ingredients. Detail regarding the purpose of ingredients in food processing definitions can vary while others proposed are more specific, such as the classification of meat that has been suggested by the American Meat Science Association Lexicon (Seman et al. 2018) and the Meat Institute Guide to Meat Processing (McCullough 2025). Ultra-processed foods (UPF) are considered the classification that is associated with the most processing applied to a particular food product, however, no standard, agreed upon definition currently exists to define the threshold of a UPF. There is considerable variation in descriptions which has resulted in confusion about which foods are UPF, with many using ‘industrial formulation’ descriptors. It is implied that food products considered to be an UPF are less healthy with high energy and/or reduced nutritional quality (McCullough 2025). The goal of this paper is to explore the complexity of UPF systems and definitions currently available for application.

## Processed Food Classification Systems

Nutrition research has continued to focus on exploring chronic health risk due to UPF consumption, especially since 2009 when the NOVA (not an acronym) food processing classification system was introduced (Monteiro 2009) to explain the connection between food, nutrition and health outcomes (Monteiro et al. 2019). This system was intended to group “foods according to the nature, extent and purpose of the industrial processing they undergo” (Monteiro et al. 2019). Recent attention on UPF’s in the food supply chain also coincide with review of dietary policy. For example, as part of dietary policy consideration, Hess et al. (2023) argued that the 2025 Dietary Guidelines Advisory Committee (DGAC) would be faced with the challenge of defining UPF’s from the published literature. Subsequently, the 2025 Scientific Report of the DGAC stated that UPF “definitions provided by authors of publications used in the DGAC literature review were used, which led to inconsistency among definitions.” The 2025 DGAC further stated that “despite this inconsistency, most of the foods categorized as ultra-processed were higher in saturated fat, sodium, and added sugars, as well as other food additives and preservatives” (DGAC 2025). Consistent increases in consumption of foods with added sugar, starch and fats correspond to increased caloric intake further impacting obesity development. The 2025 DGAC UPF characterization is similar to the summarized UPF description that Monteiro et al. (2019) provided, stating that UPF’s are “typi­cally high-energy-dense products, high in sugar, unhealthy fats and salt, and low in dietary fibre, protein, vitamins and minerals,” intended to call attention to foods with inferior nutrient composition and excessive energy. However, it is unclear whether the use of ‘low’ and ‘high’ in this context follows a definition with a specific threshold, such as the U.S. Food and Drug Administration or U.S. Department of Agriculture Food Safety Inspection Service (USDA-FSIS) classifications of ‘low’ and ‘high’ for Nutrition Facts Labeling (based on the amount of a nutrient on a per serving basis compared to the percent daily value for that nutrient) or whether these follow a standardized cutoff per serving size.

Table 1 provides an overview of category names and definitions utilized in global food processing classification systems cited in published work. Of the available food processing classification systems, NOVA is one of the most utilized classification approaches in studies evaluating chronic health outcomes and UPF consumption (Hess et al. 2023). Although some ‘processed’ categories in other systems include similar language to the NOVA 4 UPF category, NOVA specifically uses UPF as a category (Table 1). Consequentially, NOVA is perhaps the most popular classification system cited in UPF research globally (Monteiro et al. 2018; Hess et al. 2023), despite limitations of inconsistent interpretation of grouping foods and application of use that have been described in published literature (Gibney et al. 2017).

**Table 1.** Food processing classification systems and definitions adapted from Crino et al. (2017), Moubarac et al. (2014), and de Araújo et al. (2022)

Classification system	Category Name	Definition Published in Scientific Literature
NOVA Classification System (Monteiro et al. 2016)	NOVA 1: Unprocessed or minimally processed foods	Defined as minimally processed foods that may include cleaning, removal of inedible parts, grinding, drying, fermentation, pasteurization, cooling or freezing without added oils, fats, sugar, salt or other substances.
	NOVA 2: Processed culinary ingredients	Defined as ingredients made from natural foods by pressing, grinding, crushing, pulverizing or refining to produce items such as oils, fats, salt or sugar.
	NOVA 3: Processed foods	Defined as NOVA 2 ingredients added to NOVA 1 foods to preserve or increase palatability of the product.
	NOVA 4: Ultra-processed foods	Defined as industrial formulations containing little to no whole food(s).
International Agency for Research on Cancer—European Prospective Investigation into Cancer and Nutrition (IARC-EPIC; Slimani et al. 2009, Chajes et al. 2011)	Non-processed	Defined as foods which are consumed raw without further processing and/or preparation except from squeezing, cutting or washing.
	Modestly/moderately processed	Defined as: 1) commercial foods involving relatively moderate processing and consumed with no further cooking; or 2) foods processed at home and prepared/cooked from raw or moderately processed foods.
	Processed	Defined as Industrially prepared foods involving a relatively high degree of processing requiring no to minimal domestic preparation apart from heating and cooking. The classification can be divided into processed basic/daily used foods vs. highly processed foods.
International Food Information Council Foundation (IFIC; Eicher Miller et al. 2012)	Minimally processed	Defined as foods that require little processing and that maintain most of the inherent properties.
	Foods processed for preservation	Defined as foods processed to enhance nutrient composition, and increase peak freshness/preservation.
	Mixtures of combined ingredients	Defined as foods containing sweeteners, oils, spices, flavors, colors, and preservatives for collective food safety, taste/flavor, and visual appeal properties. This classification can be divided into packaged/jarred mixtures vs. mixtures likely prepared at home.
	Ready-to-eat processed	Defined as foods needing minimal to no preparation. This classification can be divided into packaged ready to eat foods vs. mixtures that may be store prepared.
	Prepared foods/meals	Defined as foods packaged for ease/speed of preparation, and freshness.
National Institute of Public Health in Mexico (NIPH; González-Castell et al. 2007)	Modern industrialized	Defined as foods incorporated into the Mexican diet as either a single product or combined with other ingredients that would be impossible to separate within the product.
	Industrialized traditional	Defined as foods that have been part of the traditional Mexican food culture according to customs/traditions but are now produced at large scale, industrial scale.
	Non-industrialized	Defined based on sub-categories below.
	3.1 Modern preparations outside the home	Defined as preparations with/ingredients that are not typical of Mexican cuisine.
	3.2 Traditional preparations	Defined as locally produced or at homefoods prepared with mixed ingredients that are often impossible to separate (ex. stews) that are part of the traditional food culture of Mexico.
	3.3 Locally made ­traditional foods	Defined as home-made or small scale foods that would be considered typical Mexican cuisine.
	3.4 Not processed	Defined as non-processed raw foods (other than steps used to select, collect or clean foods).
International Food Policy Research Institute (IFPRI; Asfaw 2011)	Unprocessed	No definition identified beyond classification.
	Primary/partially processed	No definition identified beyond classification.
	Highly processed	Defined as foods with secondary processing application into an edible form that are likely to contain high levels of added salt, fats or sugars.
UNC System (Poti et al. 2015)	Unprocessed foods	Defined as single ingredient foods that have no to very slight modifications that have not changed the inherent, original food properties and are without added ingredients. Allowable modifications may include cleaning, portioning,packaging, fat reduction or trimming, drying, pasteurization, chilling or freezing.
	Basic processed foods	Defined as single foods that may have canning, extraction, pressing, clarification, purification, milling or refining applied.
	Moderately processed foods	Defined as foods with additives/ingredients (i.e., salt, sweeteners, fats or flavors) added to single foods for flavor enhancement.
	Highly Processed Foods	Defined as multi-ingredient foods comprised of industrially formulated mixtures.
Fsanz System (Food Standards Australia New Zealand, Food Standards Code 2014)	Not Processed	No definition identified beyond classification.
	Processed	Defined as any treatment applied to food to modify from the original food property state which does not include basic separating, mincing/grinding, peeling, cutting, freezing, packing, etc.

## Challenges of Ultra-Processed Food Classification in Meat

Two primary challenges with UPF food classification still exist. First, definitions of UPF in published literature and the UPF definition in NOVA 4 point to foods that are ‘industrially formulated.’ Second, most UPF definitions in published literature reference high energy, and increased salt, sugar, fat and number of ingredients. However, both points leave users open to interpretation, which may be inconsistent among studies. Use of the term ‘formulation’ is not standardized across the food industry or among researchers, likely leading to misclassification or broad classification of foods. Because the literature doesn’t commonly describe the concentration of salt, sugar, and fat on a per gram or per serving size basis that is needed to correctly classify a food as an UPF, an opportunity for misclassification likely exists. Similarly, general descriptors such as high energy without describing a cutoff of kcal per gram or serving size may complicate the ability to consistently classify foods as UPF.

Definitions that reference ingredient list with UPF classification may likely associate number of ingredients as ‘additives’ or ‘preservatives.’ Numerous ingredients included in multi-ingredient foods provide functional properties. Simple examples include antimicrobials added to meat products to reduce bacterial growth or ingredients added that promote optimal texture in food products. Ingredients added as natural flavoring to food products may not negatively influence the nutrient profile or increase risk of developing chronic disease. For example, ground beef is often a single ingredient food (ie, beef) that has been ground, meeting the definition of NOVA 1 minimally processed foods. However, if ground beef label ingredients include beef and natural flavoring, this should not constitute classifying the product as an UPF since the USDA-FSIS allows spices (such as black pepper, basil and ginger), spice extracts, essential oils, oleoresins, onion powder, garlic powder, celery powder, onion juice and garlic juice to be added under the natural flavoring nomenclature (USDA-FSIS 2013). In meat applications, essential oils are unlikely to substantially change the nutrient profile of the product.

Gibney et al. (2017) argues that foods that have been classified as UPF according to the NOVA system may be nutritionally enhanced or fortified, particularly with nutrients that may be of concern for certain population sub-groups. As a result, fortification is intended to positively address nutrient or health concerns, which is contrary to implied negative health outcomes suggested from UPF consumption. These authors also argue that studies that report significant increases of fat, saturated fat or sodium (represented as percentage of contribution to energy intake) as UPF intake increases, may actually represent marginal effects on public health targets and UPF’s are an important source of micronutrient intake. Despite controversial debate about meat in the diet, ample evidence exists demonstrating the micronutrient density in minimally and further processed meat products. This consideration aligns with recommendations from the DGA to incorporate nutrient dense foods into dietary patterns while limiting nutrients of concern. Evaluating the nutrient density, potential perceived public health concerns and challenges with accurately assessing usual intake of all foods, including minimally processed and further processed meat, across the population are essential considerations for studies exploring chronic health risk changes due to diet.

## Conclusion

The NOVA system is the most widely utilized method for classifying food when assessing UPF and chronic health risk globally. Definitions provided in individual studies and among the NOVA 4 UPF category intend to describe the common characteristics apparent among UPF’s that include foods that generally may have higher levels of fat, sugar, and salt, increased number of ingredients used to produce the food, and perceived as an industrially formulated food. However, the lack of specificity of how much fat, sugar and salt per serving constitutes a UPF, if more than a single ingredient in a food equates to a UPF and the variability of what industrially formulated could mean, likely leaves users open for increased interpretation and increased risk of misclassification. Challenges with immediately criticizing foods with more than one ingredient include potentially overlooking the purpose of functional ingredients, micronutrients delivered via UPF intake, and enhancing flavor without increasing negative nutrients like salt, fat or sugar. As nutrition research continues to evaluate chronic health risk and UPF intake, particularly in minimally processed and further processed meat products, opportunity exists to use UPF definitions and classification as a framework to develop a more accurate method of better classifying products on the basis of nutrient density relative to established cutoffs of salt, sugar, fat and energy per serving size to systematically identify UPF’s that truly include excessive energy and limited essential, beneficial nutrients for use in exploration of UPF and chronic health risk studies. The 2025 DGAC Scientific Report highlighted the variability of UPF definitions used while simultaneously addressing the need for more research on UPF’s (well-defined) and chronic health risk.

## Author Biography

**Dr. Cody Gifford** is an Assistant Professor in the Department of Animal Science at the University of Wyoming. He earned B.S., M.S. and Ph.D. degrees from Colorado State University, inclusive of an M.S. in Animal Sciences and an M.S. in Food Science and Human Nutrition. Dr. Gifford’s research focuses on the impacts of live animal and postmortem applications on eating quality and shelf-life, and the role of meat in human dietary patterns. He serves on the counsel of scientific advisors for the Global Cold Chain Foundation in addition to serving on state and regional meat supply chain advisory committees.

Corresponding author: Cody.Gifford@uwyo.edu

## References

[vfaf048-B13] Asfaw A. 2011. Does consumption of processed foods explain disparities in the body weight of individuals? The case of Guatemala. Health Econ. 20(2):184-95. doi:10.1002/hec.1579 20029821

[vfaf048-B1] de Araújo T. P. , de Moraes M. M., Afonso C., Santos C., Rodrigues S. S. P. 2022. Food processing: comparison of different food classification systems. Nutrients. 14(4):729. doi:10.3390/nu14040729 35215379 PMC8877594

[vfaf048-B18] Chajès V. , Biessy C., Byrnes G., Deharveng G., Saadatian-Elahi M., Jenab M., Peeters P. H., Ocké M., Bueno-de-Mesquita H. B., Johansson I. et al. 2011. Ecological-level associations between highly processed food intakes and plasma phospholipid elaidic acid concentrations: results from a cross-sectional study within the European prospective investigation into cancer and nutrition (EPIC). Nutr. Cancer. 63(8):1235-50. doi:10.1080/01635581.2011.617530 22043987

[vfaf048-B2] Crino M. , Barakat T., Trevena H., Neal B. 2017. Systematic review and comparison of classification frameworks describing the degree of food processing. Nutr Food Technol. 3(1):1–12. doi:10.16966/2470-6086.138

[vfaf048-B3] DGAC. 2025. U.S. Department of Health and Human Services and U.S. Department of Agriculture. Scientific Report of the 2025 Dietary Guidelines Advisory Committee. 1st Edition, February 2025. Available online: https://www.dietaryguidelines.gov/sites/default/files/2024-12/Scientific_Report_of_the_2025_Dietary_Guidelines_Advisory_Committee_508c.pdf (accessed on July 20, 2025).

[vfaf048-B14] Eicher-Miller H. A. , Fulgoni V. L., Keast D. R. 2012. Contributions of processed foods to dietary intake in the US from 2003-2008: a report of the Food and Nutrition Science Solutions Joint Task Force of the Academy of Nutrition and Dietetics, American Society for Nutrition, Institute of Food Technologists, and International Food Information Council. J Nutr. 142(11):2065S-2072S. doi:10.3945/jn.112.164442 22990468 PMC3593301

[vfaf048-B12] Food Standards Australia New Zealand. 2014. Australia New Zealand Food Standards Code - Standard 3.2.2 - Food Safety Practices and General Requirements 2014. Available online: https://www.foodstandards.gov.au/sites/default/files/publications/SiteAssets/Pages/safefoodaustralia3rd16/Standard%203.2.2%20Food%20Safety%20Practices%20and%20General%20Requirements.pdf (accessed on September 8, 2025).

[vfaf048-B4] Gibney M. J. , Forde C. G., Mullally D., Gibney E. R. 2017. Ultra-processed foods in human health: a critical appraisal. Am. J. Clin. Nutr. 106(3):717–724. doi:10.3945/ajcn.117.160440. Epub 2017 Aug 9. Erratum in: Am J Clin Nutr. 107(3):482-483. doi:10.1093/ajcn/nqx068 28793996

[vfaf048-B15] González-Castell D. , González-Cossío T., Barquera S., Rivera J. A. 2007. Contribution of processed foods to the energy, macronutrient and fiber intakes of Mexican children aged 1 to 4 years. Salud. Publica. Mex. 49(5):345-56. doi:10.1590/s0036-36342007000500005 17952242

[vfaf048-B5] Hess J. M. , Comeau M. E., Casperson S., Slavin J. L., Johnson G. H., Messina M., Raatz S., Scheett A. J., Bodensteiner A., Palmer D. G. 2023. Dietary guidelines meet nova: Developing a menu for a healthy dietary pattern using ultra-processed foods. J. Nutr. 153(8):2472–2481. 37356502 10.1016/j.tjnut.2023.06.028

[vfaf048-B6] McCullough K. 2025. A guide to meat processing [white paper]. Meat Institute. https://www.meatinstitute.org/sites/default/files/documents/GuidetoMeatProcessing_final.pdf (accessed on July 29, 2025).

[vfaf048-B7] Monteiro C. A. 2009. Nutrition and health. 2009. The issue is not food, nor nutrients, so much as processing. Public Health Nutr. 12(5):729–731. doi:10.1017/S1368980009005291 19366466 10.1017/S1368980009005291

[vfaf048-B16] Monteiro C. A. Cannon G. Levy R. B. Moubarac J. C. Jaime P. Martins A. P. Canella D. Louzada M. L. and Parra D. 2016. NOVA. The star shines bright. World Nutr. 7:28–40.

[vfaf048-B30] Monteiro C. A. Cannon G. Moubarac J. C. Levy R. B. Louzada M. L. C. and Jaime P. C. 2018. The UN Decade of Nutrition, the NOVA food classification and the trouble with ultra-processing. Pub. Health Nutr. 21(1):5–17. doi:10.1017/S1368980017000234 28322183 PMC10261019

[vfaf048-B8] Monteiro C. A. , Cannon G., Levy R. B., Moubarac J. C., Louzada M. L., Rauber F., Khandpur N., Cediel G., Neri D., Martinez-Steele E. et al. 2019. Ultra-processed foods: what they are and how to identify them. Public Health Nutr. 22(5):936–941. doi:10.1017/S1368980018003762 30744710 10.1017/S1368980018003762 PMC10260459

[vfaf048-B9] Moubarac J. C. , Parra D. C., Cannon G., Monteiro C. A. 2014. Food classification systems based on food processing: Significance and implications for policies and actions: a systematic literature review and assessment. Curr. Obes. Rep. 3(2):256–272. doi:10.1007/s13679-014-0092-0 26626606 10.1007/s13679-014-0092-0

[vfaf048-B17] Poti J. M. , Mendez M. A., Ng S. W., Popkin B. M. 2015. Is the degree of food processing and convenience linked with the nutritional quality of foods purchased by US households? Am. J. Clin. Nutr. 101(6):1251-62. doi:10.3945/ajcn.114.100925 25948666 PMC4441809

[vfaf048-B19] Slimani N. , Deharveng G., Southgate D. A., Biessy C., Chajès V., van Bakel M. M., Boutron-Ruault M. C., McTaggart A., Grioni S., Verkaik-Kloosterman J. et al. 2009. Contribution of highly industrially processed foods to the nutrient intakes and patterns of middle-aged populations in the ­European Prospective Investigation into Cancer and Nutrition study. Eur. J. Clin. Nutr. 63(Suppl 4):S206-25. doi:10.1038/ejcn.2009.82 19888275

[vfaf048-B10] Seman D. L. , Boler D. D., Carr C., Dikeman M. E., Owens C. M., Keeton J. T., Pringle T., Sindelar J. J., Woerner D. R., de Mello A. S. et al. 2018. Meat science lexicon. Meat and Muscle Biology. 2(3):1–15. doi:10.22175/mmb2017.12.0059

[vfaf048-B11] USDA-FSIS. 2013. USDA Food Safety and Inspection Service, Natural Flavors on Meat and Poultry Labels. https://www.fsis.usda.gov/food-safety/safe-food-handling-and-preparation/food-safetybasics/natural-flavors-meat-and-poultry. (Accessed 12 September 2025).

